# Health Literacy, Service Readiness, and Community Reinforcement of Rabies-Prevention Behaviors in Rural Thailand

**DOI:** 10.3390/ijerph23040515

**Published:** 2026-04-17

**Authors:** Jinda Khumkaew, Aree Butsorn, Putthikrai Pramual

**Affiliations:** 1 College of Medicine and Public Health, Ubon Ratchathani University, Ubon Ratchathani 34190, Thailand; jinda@scphub.ac.th; 2 Si Sa Ket Provincial Public Health Office, Si Sa Ket 33000, Thailand; putthikrai.pramual@gmail.com

**Keywords:** rabies, health literacy, enabling factors, reinforcing factors, preventive behaviors, structural equation modeling, One Health, Thailand

## Abstract

**Highlights:**

**Public health relevance—How does this work relate to a public health issue?**

- Dog-mediated rabies remains a preventable rural public health threat.
- This study examines multilevel determinants of rabies-prevention behavior in high-risk Thai communities.

**Public health significance—Why is this work of significance to public health?**

- The study moves beyond descriptive KAP findings by testing a theory-based SEM.
- A key finding is that service readiness was associated with preventive behavior mainly through community reinforcement rather than through a direct pathway.

**Public health implications—What are the key implications or messages for practitioners, policy makers and/or researchers in public health?**

- Rabies programs should combine rabies-specific health literacy strengthening with community-level reinforcement, referral support, and locally coordinated follow-up.
- Feasible low-cost actions include village reminder systems, bite referral checklists, dog-vaccination outreach, and coordination among public health, livestock, and local administrative teams.

**Abstract:**

**Background:** Rabies is almost invariably fatal once clinical symptoms develop, yet it is preventable through canine vaccination and timely post-exposure prophylaxis (PEP). In rural Thailand, preventive behaviors likely depend on health literacy and contextual conditions that enable and reinforce protective action, but structural pathways remain unclear. **Methods:** We conducted a cross-sectional study among 750 adults in rabies-risk areas of Si Sa Ket Province, Thailand. A socio-ecological, One Health-informed structural equation model (SEM) examined associations among rabies-related health literacy skills (HLskill), service/system enabling conditions (ENAB), reinforcing community mechanisms (COMM), and rabies-prevention behaviors (BEHAV). **Results:** Model fit was acceptable (CFI = 0.948; TLI = 0.918; SRMR = 0.047; scaled RMSEA = 0.090). HLskill and COMM showed direct associations with BEHAV (β = 0.352 and 0.371, respectively), while ENAB was strongly associated with COMM (β = 0.939), indicating an indirect pathway through community reinforcement (β = 0.348; 95% CI [0.273, 0.424]). **Conclusions:** Rabies-prevention behaviors were associated with health literacy skills and reinforcing community mechanisms; service readiness operated primarily through community reinforcement. Rabies control should combine health literacy strengthening with community communication, coordinated dog vaccination, bite management, and timely PEP uptake.

## 1. Introduction

Rabies is a zoonotic disease with an almost 100% case-fatality once clinical symptoms appear; however, it is preventable through high dog-vaccination coverage and timely administration of human post-exposure prophylaxis (PEP) [1,2]. Despite decades of global and national progress, including the Zero by 30 initiative under a One Health approach, dog-mediated rabies remains a major public health problem. It continues to cause an estimated 59,000 human deaths annually, particularly in Asia and Africa, with substantial social and economic burden. Transmission most commonly occurs through exposure to infectious saliva, especially through dog bites, and the disease is almost invariably fatal once clinical symptoms develop. Therefore, key control strategies emphasize mass dog vaccination, timely access to post-exposure prophylaxis (PEP), strengthened surveillance, intersectoral coordination, and management of free-roaming dog populations [3].

Thailand continues to report animal rabies and sporadic human deaths, despite national availability of vaccines and biologics. Laboratory surveillance consistently identifies dogs as the principal infected species, with spatial analyses revealing persistent transmission foci associated with environmental and social factors [4]. Human rabies deaths tend to co-locate with animal-positive areas [5]. Within Health Region 10, Si Sa Ket Province represents a high-priority setting: provincial records documented 24 laboratory-confirmed rabid animals between 2020 and 2025—predominantly owned or free-roaming dogs with low vaccination coverage among positives—and at least one human rabies death in 2025 [6]. While local initiatives demonstrate the potential of community-participatory surveillance and prevention under a One Health framework, substantial variability in district-level implementation suggests that service readiness does not always translate into routine preventive practice [7,8].

At the household level, studies from Asia and Africa consistently link rabies-prevention practices to knowledge and attitudes among dog owners and residents in high-incidence areas [7,8,9]. However, the translation of knowledge into action depends not only on awareness but also on individuals’ health literacy (HL) capacities and on how services are organized, delivered, and reinforced within communities [8,9,10]. A key limitation of the existing literature is that most knowledge–attitude–practice (KAP) studies describe associations without testing the mechanisms through which service or system readiness—such as vaccine availability, outreach activities, and case management—is converted into sustained preventive behaviors. Structural barriers including distance to services, waiting times, vaccine availability, and household costs continue to impede timely PEP uptake and annual dog vaccination in rural settings [3,11].

Integrated Bite Case Management (IBCM), a practical One Health intervention, has been shown to improve case detection, risk assessment, and appropriate PEP use through coordinated human–animal health workflows [12,13]. Nevertheless, its effectiveness is likely contingent on community- and policy-level platforms—such as local regulations, municipal support, and village-based campaigns—that translate service readiness into household action and reinforce routine protective behaviors. From a health education and health promotion perspective, these observations underscore the need to move beyond descriptive KAP surveys toward theory-informed, multilevel models that explicitly integrate individual HL skills, service/system enabling conditions, and reinforcing community mechanisms.

Guided by socio-ecological theory and the PRECEDE–PROCEED framework, and aligned with a One Health perspective, we conceptualized rabies-prevention behavior as being associated with interacting predisposing, enabling, and reinforcing determinants [14,15,16,17]. We hypothesized that service/system-level enabling factors (ENAB) would be associated with household rabies-prevention behaviors (BEHAV) primarily through reinforcing community mechanisms (COMM), such as local governance support, social norms, and collective action. In parallel, individual rabies-related health literacy skills—encompassing the abilities to access, understand, appraise, and apply relevant information (HLskill)—were expected to show a direct positive association with preventive behavior, consistent with functional, interactive, and critical health literacy theory [10].

Study aim. To address these gaps in a high-priority Thai province with documented rabies transmission, this study specified and tested a socio-ecological structural equation model (SEM) integrating HLskill, ENAB, and COMM as multilevel determinants of rabies-prevention behaviors among adults residing in laboratory-defined rabies-risk areas of Si Sa Ket Province, with data collected in 2025. Specifically, the study aimed to: (i) describe household rabies-prevention behaviors; (ii) examine associations among health literacy skills, enabling conditions, community reinforcement mechanisms, and preventive behaviors; and (iii) evaluate a theoretically informed SEM specifying direct associations of HLskill and COMM with behavior and an indirect ENAB → COMM → BEHAV pathway, to inform rabies-focused health promotion strategies in rural Thailand.

## 2. Materials and Methods

### 2.1. Study Design and Setting

An analytical cross-sectional study was conducted in Si Sa Ket Province, northeastern Thailand. Si Sa Ket was selected as a high-priority setting based on provincial surveillance records documenting ongoing rabies transmission, including laboratory-confirmed animal rabies cases and sporadic human rabies deaths. For this study, rabies-risk areas were operationally defined as subdistricts or villages reporting at least one laboratory-confirmed rabid animal or at least one human rabies case during 2020–2024, in accordance with provincial surveillance protocols. Data collection was conducted between August and October 2025 within these defined risk areas, to enhance the ecological validity of observed rabies-prevention behaviors and their determinants.

### 2.2. Participants and Sampling

#### 2.2.1. Eligibility Criteria

Eligible participants were community-dwelling adults aged 18 years or older who had resided in the selected villages for at least six months prior to data collection. Individuals were excluded if they were unable to provide informed consent, for example due to severe cognitive impairment assessed by trained interviewers, or if they were unable to communicate adequately in Thai or Isan to complete the interview.

#### 2.2.2. Sampling Procedure

A multistage cluster sampling strategy was employed.

In the first stage, districts and subdistricts meeting the rabies-risk definition were selected from the provincial surveillance registry. In the second stage, villages within selected subdistricts were sampled using probability proportional to size (PPS) sampling where population data were available; otherwise, simple random sampling was applied. In the third stage, households within each selected village were systematically sampled using a random start and fixed interval. One eligible adult per household was selected using a Kish grid or next-birthday method to minimize interviewer selection bias.

If a selected household or respondent was unavailable or declined participation, up to two revisits were attempted at different times or on different days before replacement using the same sampling interval.

#### 2.2.3. Sample Size Considerations

A target sample size of 750 participants was specified a priori to support both the multistage cluster survey design and stable estimation for the planned structural equation modeling (SEM). This sample size was set above the minimum for a simple random sample to account for clustering and possible within-village correlation. For SEM, sample adequacy was considered in relation to model complexity, the retained latent constructs, and the need for stable parameter estimates, reliable standard errors, and adequate statistical power. The target of 750 participants exceeded commonly cited minimum recommendations for applied SEM [18] and also allowed for limited item non-response while preserving analytic stability under robust estimation. The final analytic sample (*n* = 750) was therefore considered appropriate for both the survey design and the planned analyses.

#### 2.2.4. Weighting and Clustering

Given the multistage sampling design, analyses accounted for clustering at the village level using cluster-robust standard errors. Where selection probabilities differed materially, sampling weights were calculated and examined in sensitivity analyses; substantive results were unchanged, and unweighted estimates with robust standard errors are presented.

### 2.3. Instrument Development and Measures

A structured questionnaire was developed from international and national rabies-prevention guidance and was conceptually informed by socio-ecological theory, the PRECEDE–PROCEED framework, and a One Health perspective. The full questionnaire comprised five sections: (1) sociodemographic characteristics, (2) animal exposure and rabies-related experience, (3) rabies-related health literacy, (4) rabies-prevention behavior, and (5) community and service conditions relevant to animal control and rabies prevention. Section 1 included 6 sociodemographic items used for sample description. Section 2 comprised 11 items on pet ownership, animal exposure, bite or scratch experience, vaccination history, and rabies-related information exposure, and was used for contextual description. Section 3 contained 25 items: 10 dichotomous knowledge items and 15 health-literacy skill items measured on a 5-point Likert-type scale. Section 4 included 10 household rabies-prevention behavior items measured on a 3-point behavioral frequency scale. Section 5 comprised 23 items assessing community and service context relevant to rabies prevention, including enabling and reinforcing conditions, measured on a 5-point Likert-type scale.

For SEM analysis, HLskill was derived from Section 3A and 3B (rabies-related knowledge and health-literacy skills), BEHAV from Section 4 (household rabies-prevention behavior), and ENAB and COMM from Section 5 (community and service context). The final SEM retained theory-consistent composite indicators representing these constructs, as summarized in Supplementary Tables S1 and S2.

Response formats varied by section and construct. Knowledge items were scored dichotomously (correct = 1, incorrect = 0). Health-literacy skill items and community/service-context items were measured on 5-point Likert-type scales. Household behavior items were measured on a 3-point behavioral frequency scale, with higher scores indicating stronger preventive practice.

For interpretability, health literacy scores were linearly transformed to a 0–10 metric using the following formula: Rescaled score = [(observed mean score − minimum possible score) / (maximum possible score − minimum possible score)] × 10. Higher values indicate stronger rabies-related health literacy skills. This transformation was applied solely to improve interpretability and did not alter the relative ordering of respondents.

An English rendering of the questionnaire structure, including response formats, scoring notes, and construct mapping, is provided in Supplementary Table S1.

### 2.4. Validity and Reliability

Content validity was reviewed by experts in public health, veterinary public health, and community health systems. Internal consistency of multi-item domains was evaluated using Cronbach’s alpha. Construct validity of the retained SEM constructs was evaluated using confirmatory factor analysis. Convergent validity was assessed using standardized factor loadings, composite reliability (CR), and average variance extracted (AVE), whereas discriminant validity was examined using the Fornell–Larcker criterion. Detailed measurement properties are reported in Supplementary Table S2.

### 2.5. Statistical Analysis

Descriptive statistics were used to summarize sample characteristics and study variables. The measurement model was evaluated using confirmatory factor analysis (CFA) with robust maximum likelihood estimation to reduce sensitivity to non-normality. The structural model specified direct paths from HLskill and COMM to BEHAV and an indirect pathway from ENAB to BEHAV through COMM.

Model fit was assessed using multiple indices, including the comparative fit index (CFI), Tucker–Lewis index (TLI), standardized root mean square residual (SRMR), and root mean square error of approximation (RMSEA). Standardized path coefficients (β) with robust standard errors were reported. Indirect effects were evaluated using bias-corrected bootstrapping (1000 iterations).

Missing data were minimal and handled using full-information maximum likelihood under a missing-at-random assumption. During preliminary estimation, a Heywood case was observed for one variance parameter of COMM. A Heywood case refers to an inadmissible SEM solution, such as a negative residual variance or an implausible variance estimate, and may arise from model misspecification, empirical overlap among indicators, or sampling-related instability. In the present model, a minor Heywood case was addressed by constraining the relevant residual variance to a small positive value (0.001), after which model admissibility was restored and the substantive conclusions remained unchanged. All analyses were conducted using the SEM module in jamovi (lavaan backend). Because the SEM used domain-level composite indicators derived from multiple questionnaire items, these indicators were treated as approximately continuous for model estimation.

## 3. Results

### 3.1. Sample Characteristics

A total of 750 adults participated in the study. The majority were female (77.9%), with a mean age of 52.8 years (SD = 10.1). This sex distribution likely reflects household-based daytime interviews in rural communities, where women were more often available at the time of data collection. For descriptive purposes, age was categorized into three groups: 18–39 years (9.3%), 40–59 years (68.8%), and ≥60 years (21.9%). Most participants were married or cohabiting (74.7%) and engaged primarily in agricultural occupations (79.2%).

Monthly household income was modest, with a mean of 5017.9 THB (SD = 5354.7). For parsimony and given the skewed income distribution, income was categorized into two groups: ≤5000 THB per month (58.4%) and >5000 THB per month (41.6%). Approximately 72.2% of respondents reported keeping dogs and/or cats.

One in five participants (20%) reported experiencing an animal bite or scratch within the previous 12 months; more than half of these incidents involved the respondent’s own animal. Among those exposed, most sought care within 1–2 days, and 94.2% of individuals who initiated post-exposure prophylaxis completed the full vaccination schedule.

Mean rabies-related health literacy was relatively high (mean = 7.64 out of 10, SD = 1.45). Preventive behaviors were also generally high, with 74.5% of respondents reporting consistent engagement in recommended rabies-prevention practices (mean = 2.71, SD = 0.53). Detailed demographic and exposure characteristics are presented in Table 1.

### 3.2. Measurement Model

Confirmatory factor analysis supported the retained measurement structure. All retained standardized factor loadings were statistically significant (*p* < 0.001) and ranged from 0.710 to 0.923 for the multi-indicator latent constructs. Cronbach’s alpha values were 0.666 for HLskill, 0.793 for ENAB, and 0.825 for COMM; the corresponding CR values were 0.678, 0.807, and 0.852, and the AVE values were 0.511, 0.585, and 0.735, respectively. These findings supported acceptable internal consistency and convergent validity for the retained constructs in an applied SEM context. Detailed measurement-model results are provided in Supplementary Table S2.

Discriminant validity was assessed using the Fornell–Larcker criterion. The criterion was acceptable for most retained constructs, although it was not fully met between ENAB and COMM, suggesting partial conceptual overlap in this rural context. BEHAV was modeled as a single observed composite indicator in the final SEM; therefore, CR and AVE are not presented in the same manner as for the multi-indicator latent constructs. Taken together, these findings indicate that the measurement model largely supported the underlying theoretical constructs, with the noted exception of discriminant validity between ENAB and COMM.

### 3.3. Structural Model

The structural equation model demonstrated acceptable overall fit to the data (χ^2^(18) = 127, CFI = 0.948, TLI = 0.918, SRMR = 0.047, scaled RMSEA = 0.090). Although the RMSEA slightly exceeded conservative cut-offs, this index may over-penalize models with relatively small degrees of freedom. We therefore interpreted model adequacy using multiple fit indices, including CFI, TLI, and SRMR, together with theoretical coherence, which supported practical adequacy for an applied public health model.

As hypothesized, both rabies-related health literacy skills and reinforcing community mechanisms showed significant direct positive associations with rabies-prevention behaviors. Specifically, higher HLskill was associated with more consistent preventive behavior (β = 0.352, *p* < 0.001), and stronger community reinforcement mechanisms were similarly associated with improved preventive practices (β = 0.371, *p* < 0.001).

Service/system enabling conditions exhibited a strong positive association with reinforcing community mechanisms (β = 0.939, *p* < 0.001). This pattern indicates a substantial indirect pathway from enabling conditions to preventive behavior through community reinforcement. The indirect effect (ENAB → COMM → BEHAV) was estimated at β = 0.348 (SE = 0.038; 95% BC bootstrap CI [0.273, 0.424]; *p* < 0.001), confirming the statistical significance of this mediated pathway. Standardized path coefficients, standard errors, and confidence intervals are summarized in Table 2.

**Figure 1:**
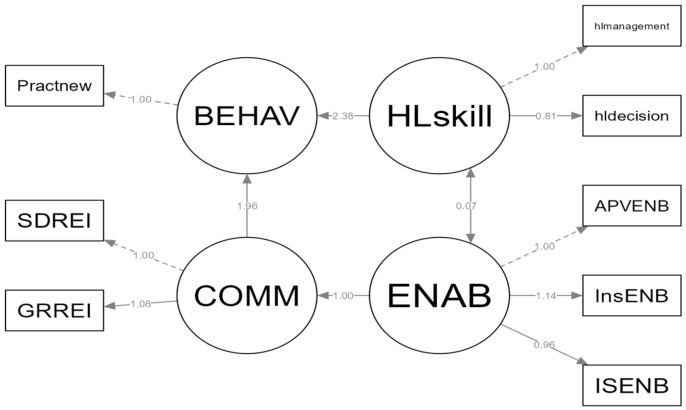
Structural equation model of rabies-prevention behavior. HLskill = rabies-related health literacy skills, including accessing, understanding, appraising, and applying rabies-prevention information in daily decision-making; ENAB = enabling or service-readiness conditions, including practical access to vaccination, information, and local service support; COMM = reinforcing community mechanisms, including local communication, encouragement, shared norms, and community support for rabies prevention; BEHAV = household rabies-prevention behavior, including animal vaccination practices, bite response, risk avoidance, reporting, and participation in prevention activities. Model fit: χ^2^(18) = 127, *p* < 0.001; CFI = 0.948; TLI = 0.918; SRMR = 0.047; scaled RMSEA = 0.090.

Overall, the model explained 32.6% of the variance in rabies-prevention behaviors (R^2^ = 0.326). The final structural model is illustrated in Figure 1.

## 4. Discussion

### 4.1. Principal Findings

In this rural Thai setting with documented rabies transmission, household rabies-prevention behaviors were generally high. However, the structural equation model demonstrated that preventive behavior was associated with distinct yet connected pathways operating at individual, community, and service levels. Rabies-related health literacy skills (HLskill) and reinforcing community mechanisms (COMM) both showed significant direct associations with preventive behavior, while service/system enabling conditions (ENAB) were associated with behavior primarily through a strong indirect pathway operating via community reinforcement. Taken together, these findings suggest that strengthening individual knowledge and skills alone may be insufficient unless enabling service conditions are translated into community-embedded mechanisms—such as local rules, collective mobilization, reminders, and outreach—that support routine protective practice.

### 4.2. Interpretation of Behavioral Pathways

The observed direct association between health literacy skills and rabies-prevention behavior is consistent with functional, interactive, and critical health literacy theory, which emphasizes individuals’ capacities to access, understand, appraise, and apply health information in real-world contexts [10]. In the context of rabies prevention, stronger literacy skills may support more accurate risk appraisal following animal exposure, appropriate wound management, and timely decision-making regarding PEP initiation, which is consistent with the HLskill → BEHAV association identified in this study.

These findings also align with the PRECEDE–PROCEED framework and socio-ecological models, which posit that predisposing factors (such as health literacy), enabling conditions (service/system access), and reinforcing mechanisms (community and organizational supports) work together to shape health behavior [14,15,16]. Notably, the slightly stronger association observed between reinforcing community mechanisms and preventive behavior, compared with health literacy, underscores the practical importance of community-level reinforcement in rural settings where transportation constraints, opportunity costs, and animal-handling barriers are common [12,13].

The very strong ENAB → COMM pathway further suggests that service/system readiness—such as vaccine availability, workforce capacity, and physical access to services—may be associated with household practice mainly through reinforcement at the community level. This finding extends the predominantly descriptive KAP-oriented literature by identifying a plausible pathway through which enabling conditions may be translated into routine preventive action, rather than assuming a direct link between service availability and behavior [8,9,10].

### 4.3. Service Readiness as an Indirect Pathway to Behavior and Its Practical Implications

Operationally, the indirect role of service readiness observed in this study is consistent with experiences from integrated bite case management (IBCM) initiatives in settings such as Tanzania and the Philippines. The present findings extend beyond the general observation that dog vaccination and timely PEP are important. The more informative result was that service readiness was associated with household rabies-prevention behavior mainly through community reinforcement rather than through a direct pathway. This suggests that available services alone may not be sufficient unless local actors help residents interpret risk, remember vaccination schedules, know where to seek care after bites, and receive encouragement to complete recommended actions. In this rural context, practical barriers likely include distance to service points, transport burden, uncertainty about where to seek care, difficulty handling animals for vaccination, and irregular local communication. Affordable actions include village dog registers, scheduled reminder messages, bite referral checklists, coordinated outreach vaccination, and routine engagement of community volunteers and local administrative structures. In these contexts, coordinated human–animal health workflows have strengthened detection of suspect rabid animals and supported appropriate PEP decision-making through interprofessional triage and information sharing [12,13]. Such experiences reinforce the interpretation that services are most effective when embedded within community and policy platforms that facilitate coordination and collective action, rather than functioning as stand-alone technical inputs.

Moreover, evidence that distance, time, and cost are associated with completion of PEP and uptake of dog vaccination supports the need for pragmatic service design in endemic rural settings, including streamlined care pathways and dose-sparing or intradermal regimens where consistent with national guidance [3,11]. Integrating animal-sector activities led by the Department of Livestock Development with human surveillance data from the Department of Disease Control may further strengthen spatial targeting of vaccination and outreach, consistent with prior spatial analyses of rabies transmission in Thailand [4,5]. Together, these findings align with the One Health “Zero by 30” agenda, which emphasizes coordinated, multisectoral approaches to rabies elimination [2].

### 4.4. Implications for Rabies Prevention Programs

The present findings have several implications for rabies-prevention strategies in endemic rural settings such as Si Sa Ket Province. First, strengthening rabies-specific health literacy should remain a core component of prevention programs, with emphasis on progression from functional to interactive and critical skills. Priority should be given to improving post-exposure risk appraisal, correct wound management, and timely initiation of PEP, particularly among pet-owning households and populations at higher exposure risk [10,17].

Second, program design should prioritize reinforcing community mechanisms that sustain preventive norms, including village-based campaigns, local bylaws supporting dog vaccination, and intersectoral collaboration among health, veterinary, and local government actors. In resource-constrained settings, this does not necessarily require major new infrastructure. Feasible measures include village-level reminder systems, locally maintained dog registers, bite referral checklists, designated village contacts for bite reporting, and synchronized dog-vaccination outreach using existing public health and livestock service rounds. Third, institutionalizing IBCM through formalized joint human–animal response teams, standardized reporting tools, and shared monitoring dashboards can operationalize the ENAB→COMM linkage by converting system readiness into coordinated community action [12,13].

Finally, strengthening spatial targeting and performance monitoring through subdistrict- and village-level dashboards—aligned with national reporting systems and “Zero by 30” targets—may support more efficient allocation of outreach, vaccination, and risk communication resources in higher-risk areas [4,5].

### 4.5. Strengths and Limitations

This study has several strengths. It draws on a programmatically defined sampling frame from areas with documented rabies risk, employs validated measures, and applies a theory-informed socio-ecological SEM integrating One Health perspectives. By explicitly modeling direct and indirect pathways, the analysis moves beyond descriptive KAP studies to elucidate mechanisms linking service readiness, community reinforcement, and household behavior.

Several limitations should be acknowledged. First, the cross-sectional design precludes causal inference; all reported relationships should be interpreted as associations. Second, self-reported measures may be subject to recall or social desirability bias. Third, the RMSEA slightly exceeded conservative thresholds; however, as this index can over-penalize models with small degrees of freedom, model adequacy was judged using multiple indices (CFI, TLI, SRMR) together with theoretical coherence. Fourth, a minor Heywood case was resolved by constraining the residual variance of the COMM construct to 0.001 [19,20]; substantive conclusions were unchanged. Fifth, the sample was predominantly female (77.9%), likely reflecting household-based daytime data collection in rural communities where women were more often available during interviewer visits; this may limit generalizability if rabies-related caregiving, animal management, or health-seeking behaviors differ by gender. Sixth, the inter-construct correlation between ENAB and COMM (r ≈ 0.939) exceeded the square roots of their respective AVE values (√AVE = 0.765 and 0.857; AVE = 0.585 and 0.735, respectively), indicating that discriminant validity between these constructs was not fully supported by the Fornell–Larcker criterion and suggesting potential conceptual overlap in this rural context. Seventh, HLskill was represented by only two indicators, yielding a just-identified sub-model (df = 0) and slightly subthreshold internal consistency (α = 0.666; CR = 0.678); findings involving this construct should be interpreted cautiously. Finally, the single-province sampling frame limits generalizability to settings with different health-system or community structures.

## 5. Conclusions

Rabies-prevention behaviors in Si Sa Ket Province were associated with factors operating at three connected levels: individual health literacy skills, community reinforcement, and service readiness. Health literacy skills and reinforcing community mechanisms showed direct positive associations with preventive behavior, while service readiness was associated with preventive behavior mainly through stronger community-level reinforcement. In practical terms, lower preventive performance in rural settings may reflect transport burden, uncertainty about where to seek care after bites, difficulty handling animals for vaccination, incomplete recall of vaccination schedules, and uneven local communication. Programs aiming to achieve rabies-free districts should therefore combine rabies-specific health literacy strengthening with feasible local actions such as village reminder systems, bite referral support, coordinated dog-vaccination outreach, institutionalized IBCM, and spatially targeted communication and surveillance. This multilevel strategy is consistent with One Health and “Zero by 30” goals and provides a theory-informed foundation for designing integrated rabies health-education and community health-promotion programs in rural, resource-constrained settings [2,10,12,13,16,17].

## Figures and Tables

**Table 1:** Demographic and exposure characteristics of respondents (*n* = 750).

Variable	*n*	%	Mean (SD)
Sex			
Female	584	77.9	
Male	166	22.1	
Age (years)			52.77 (10.06)
Young adult (18–39)	70	9.3	
Middle-aged (40–59)	516	68.8	
Older adult (≥60)	164	21.9	
Marital status			
Married/cohabiting	560	74.7	
Education			
Primary	282	37.6	
Secondary	417	55.6	
Above secondary	51	6.8	
Occupation			
Agriculture	594	79.2	
Daily wage/labor	74	9.9	
Trade/business	42	5.6	
Monthly income (THB)			5017.85 (5354.71)
Keeping dogs/cats			
Yes	541	72.2	
No	209	27.8	
Animal bite/scratch in past 12 months			
Yes	150	20.0	
No	600	80.0	

**Table 2:** Standardized path coefficients and indirect effect of the structural equation model.

Structural Path	Standardized β	Unstandardized *b*	SE	95% CI	*p* Value
HLskill → BEHAV	0.352	2.38	0.334	[1.722, 3.030]	<0.001
COMM → BEHAV	0.371	1.96	0.203	[1.558, 2.350]	<0.001
ENAB → COMM	0.939	1.00	0.037	[0.931, 1.080]	<0.001
ENAB → COMM → BEHAV	0.348	—	0.038	[0.273, 0.424]	<0.001

Standardized β values are shown for all paths. For direct effects, unstandardized *b*, SE, and 95% CI are reported from the SEM parameter estimates. For the indirect effect, the reported CI is based on bias-corrected bootstrap resampling (1000 iterations).

## Data Availability

The data are not publicly available due to privacy and ethical restrictions, as the dataset includes potentially sensitive human-participant information. De-identified Summary Data Supporting the reported findings are included in the manuscript and Supplementary Materials where applicable. Additional de-identified data may be made available by the corresponding author upon reasonable request and subject to ethics approval and institutional regulations.

## Supplementary Materials

The following supporting information can be downloaded at: https://www.mdpi.com/article/10.3390/ijerph23040515/s1, Table S1: English rendering of the questionnaire structure, including response formats, scoring notes, and construct mapping; Table S2: Fit indices, measurement properties, and structural path estimates for the final structural equation model (SEM).
