# Supplementary material for: Health Literacy, Service Readiness, and Community Reinforcement of Rabies-Prevention Behaviors in Rural Thailand
Source: Int J Environ Res Public Health. 2026 Apr 17;23(4):515. doi: 10.3390/ijerph23040515 (PMC13116964; doi:10.3390/ijerph23040515)
Supplement: Supplementary file 1 [file ijerph-23-00515-s001.zip › Supplementary_Table_S1.pdf]

Supplementary Table S1

The questionnaire was originally developed and administered in Thai for community-based fieldwork. This supplementary file provides an English rendering of the questionnaire structure for reporting and reproducibility purposes.

Response scale legend

5-point Likert-type scale: 5 = always / very much / strongly agree; 4 = often / much / agree; 3 = sometimes / moderate / unsure; 2 = rarely / little / disagree; 1 = never / not at all / strongly disagree, depending on item wording.

3-point behavioral frequency scale: 3 = performed regularly; 2 = performed often; 1 = performed sometimes or never.

Dichotomous knowledge items: correct = 1; incorrect = 0.

Section 1. Sociodemographic characteristics

Item	English rendering of original item/content	Response format	Scoring / analysis note
1	Sex	Categorical	Descriptive only
2	Age (years)	Fill-in / numeric	Descriptive only
3	Marital status	Categorical	Descriptive only
4	Education level	Categorical	Descriptive only
5	Occupation	Categorical	Descriptive only
6	Monthly income	Categorical / fill-in	Descriptive only

Section 2. Animal exposure and rabies-related experience

Item	English rendering of original item/content	Response format	Scoring / analysis note
1	Dog/cat ownership in the household	Binary / categorical	Descriptive only
2	Type and number of animals in the household	Categorical / fill-in / multiple response	Descriptive only
3	Vaccination status of household dogs/cats	Categorical	Contextual description
4	Frequency of rabies vaccination among owned animals	Categorical	Contextual description
5	Reasons for incomplete or absent animal vaccination	Multiple response / categorical	Contextual description
6	History of dog or cat bites/scratches	Binary	Contextual description
7	Circumstances of bite or scratch exposure	Categorical	Contextual description
8	Health service use after exposure	Binary / categorical	Contextual description
9	Completion of rabies vaccination / PEP if indicated	Categorical	Contextual description
10	Sources of rabies information received in the past 6 months	Multiple response	Contextual description
11	Awareness of laboratory-confirmed rabies cases in the community	Binary / categorical	Contextual description

Section 3A. Rabies-related knowledge and understanding

Item	English rendering of original item/content	Response format	Scoring / analysis note
1	Rabies can be fatal once symptoms develop	Dichotomous	Correct = 1; incorrect = 0
2	Rabies can be prevented	Dichotomous	Correct = 1; incorrect = 0
3	Dogs and cats can transmit rabies	Dichotomous	Correct = 1; incorrect = 0
4	Immediate wound washing reduces rabies risk	Dichotomous	Correct = 1; incorrect = 0
5	Animal vaccination helps prevent rabies transmission	Dichotomous	Correct = 1; incorrect = 0
6	Medical care should be sought promptly after bites or scratches	Dichotomous	Correct = 1; incorrect = 0
7	Traditional treatment alone cannot prevent rabies	Dichotomous	Correct = 1; incorrect = 0
8	Apparently healthy animals may still pose rabies risk	Dichotomous	Correct = 1; incorrect = 0
9	Suspected rabid animals should be reported	Dichotomous	Correct = 1; incorrect = 0
10	Annual vaccination of dogs/cats is recommended	Dichotomous	Correct = 1; incorrect = 0

Section 3B. Rabies-related health literacy skills

Item	English rendering of original item/content	Response format	Scoring / analysis note
11	Ability to access rabies-related information from health sources	5-point Likert-type scale	Higher = stronger health literacy skill
12	Ability to receive and understand information about nearby vaccination services	5-point Likert-type scale	Higher = stronger health literacy skill
13	Tendency to forget or fail to follow professional advice after exposure	5-point Likert-type scale	Reverse-coded where applicable
14	Ability to prevent owned dogs from mixing with stray dogs	5-point Likert-type scale	Higher = stronger health literacy skill
15	Ability to avoid direct contact with unowned or stray dogs	5-point Likert-type scale	Higher = stronger health literacy skill
16	Knowledge of whom to contact when a suspected rabid animal is found	5-point Likert-type scale	Higher = stronger health literacy skill
17	Tendency not to track or remember vaccination schedules	5-point Likert-type scale	Reverse-coded where applicable
18	Ability to appraise rabies information from reliable sources	5-point Likert-type scale	Higher = stronger health literacy skill
19	Tendency to share information without checking its accuracy	5-point Likert-type scale	Reverse-coded where applicable
20	Confidence in asking health personnel for clarification	5-point Likert-type scale	Higher = stronger health literacy skill
21	Difficulty paying for animal vaccination	5-point Likert-type scale	Interpreted within practical management context
22	Convenience of traveling to vaccination or health service points	5-point Likert-type scale	Interpreted within practical management context
23	Ability to make appropriate decisions after bite or scratch exposure	5-point Likert-type scale	Higher = stronger health literacy skill
24	Ability to act promptly after dog/cat bite or scratch exposure	5-point Likert-type scale	Higher = stronger health literacy skill
25	Ability to use rabies-prevention information in daily practice	5-point Likert-type scale	Higher = stronger health literacy skill

Section 4. Household rabies-prevention behavior

Item	English rendering of original item/content	Response format	Scoring / analysis note
1	Taking household dogs/cats for annual rabies vaccination	3-point behavioral frequency scale	Higher = better preventive behavior
2	Washing wounds immediately after bites or scratches	3-point behavioral frequency scale	Higher = better preventive behavior
3	Seeking medical care promptly after exposure	3-point behavioral frequency scale	Higher = better preventive behavior
4	Following professional advice regarding rabies prevention after exposure	3-point behavioral frequency scale	Higher = better preventive behavior
5	Avoiding contact with animals suspected of having rabies	3-point behavioral frequency scale	Higher = better preventive behavior
6	Reporting suspected rabid animals to relevant authorities	3-point behavioral frequency scale	Higher = better preventive behavior
7	Participating in rabies-prevention activities in the community	3-point behavioral frequency scale	Higher = better preventive behavior
8	Preventing food waste from attracting stray dogs	3-point behavioral frequency scale	Higher = better preventive behavior
9	Encouraging family members or neighbors to vaccinate animals	3-point behavioral frequency scale	Higher = better preventive behavior
10	Using recommended bite-management practices consistently	3-point behavioral frequency scale	Higher = better preventive behavior

Section 5. Community and service context relevant to rabies prevention

Item	English rendering of original item/content	Response format	Scoring / analysis note
1	Availability of dog registration systems in the community	5-point Likert-type scale	Input to ENAB / COMM context
2	Availability of vaccine storage or cold-chain support in the area	5-point Likert-type scale	Input to ENAB
3	Availability of annual rabies vaccination services for dogs	5-point Likert-type scale	Input to ENAB
4	Availability of places or systems for managing stray dogs	5-point Likert-type scale	Input to ENAB / COMM context
5	Availability of sterilization services for dogs/cats	5-point Likert-type scale	Input to ENAB
6	Presence of volunteers or responsible persons for stray dog management	5-point Likert-type scale	Input to COMM
7	Presence of local committees or teams responsible for rabies prevention	5-point Likert-type scale	Input to COMM
8	Community education or training on rabies prevention	5-point Likert-type scale	Input to COMM
9	Use of signs, announcements, or campaigns promoting rabies prevention	5-point Likert-type scale	Input to COMM
10	Community members help each other with rabies-prevention advice or support	5-point Likert-type scale	Input to COMM
11	Availability of local support or encouragement for households to act on rabies-prevention advice	5-point Likert-type scale	Input to COMM
12	Practical burden related to travel or cost when	5-point Likert-type	Contextual factor related to ENAB

Item	English rendering of original item/content	Response format	Scoring / analysis note
	obtaining vaccination or care	scale	
13	Availability of community funds or local financial support mechanisms	5-point Likert-type scale	Input to ENAB / COMM context
14	Presence of clearly responsible agencies or local structures for rabies prevention	5-point Likert-type scale	Input to ENAB / COMM context
15	Existence of village agreements or community rules supporting rabies prevention	5-point Likert-type scale	Input to COMM
16	Presence of local government announcements or regulations relevant to rabies control	5-point Likert-type scale	Input to COMM
17	Availability of covered waste bins or suitable waste management systems	5-point Likert-type scale	Environmental context
18	Practices related to feeding or attracting stray dogs in the community	5-point Likert-type scale	Environmental / community context
19	Availability of community communication channels for rabies information	5-point Likert-type scale	Input to COMM
20	Problems related to dogs scavenging waste or environmental contamination	5-point Likert-type scale	Environmental context
21	Presence of local rabies-prevention working groups	5-point Likert-type scale	Input to COMM
22	Availability of vaccines or preventive services for exposed people	5-point Likert-type scale	Input to ENAB
23	Accessibility of sterilization services for animal population control	5-point Likert-type scale	Input to ENAB

Scoring notes

1. HLskill: knowledge items and health literacy skill items were combined at domain level; the resulting composite was linearly transformed to a 0–10 scale for interpretability.

2. BEHAV: higher values indicate stronger household rabies-prevention behavior.

3. ENAB and COMM: item means were first computed within subdomains and then summarized into the retained SEM indicators.

4. Reverse-coded items were recoded before domain aggregation, where applicable.

Item grouping used for domain construction and SEM reduction

Final construct	Original item range	Intermediate domain/subdomain	Retained SEM indicator(s)
HLskill	Section 3A items 1–10; Section 3B items 11–25	Knowledge and health literacy skill domains	hlmanagement; hldecision
ENAB	Section 5 selected items	Access / institutional / vaccination-service readiness	APVENB; InsENB; ISENB
COMM	Section 5 selected items	Social support / group reinforcement	SDREI; GRREI
BEHAV	Section 4 items 1–10	Household rabies-prevention behavior composite	Practnew

Abbreviations: HLskill, rabies-related health literacy skills; ENAB, service/system enabling conditions; COMM, reinforcing community mechanisms; BEHAV, rabies-prevention behaviors; SEM, structural equation model; PEP, post-exposure prophylaxis.
