# Supplementary material for: Health Literacy, Service Readiness, and Community Reinforcement of Rabies-Prevention Behaviors in Rural Thailand
Source: Int J Environ Res Public Health. 2026 Apr 17;23(4):515. doi: 10.3390/ijerph23040515 (PMC13116964; doi:10.3390/ijerph23040515)
Supplement: Supplementary file 1 [file ijerph-23-00515-s001.zip › Supplementary_Table_S2.pdf]

Supplementary Table S2

This supplementary file presents the fit indices, measurement properties, and structural path estimates for the final structural equation model (SEM) retained for interpretation in the revised manuscript. Standardized factor loadings were obtained from the final SEM output. Cronbach's alpha was derived from reliability analyses of the corresponding scales. Composite reliability (CR) and average variance extracted (AVE) were used to assess convergent validity for the retained multi-indicator constructs. BEHAV was represented as a single observed composite indicator in the final SEM and is therefore not directly comparable to the multi-indicator latent constructs in terms of internal consistency coefficients or AVE.

A. Final SEM fit indices

Fit index	Value	Interpretation
χ^2 (df = 18)	127	A significant chi-square is common in large samples and was interpreted alongside additional fit indices.
CFI	0.948	Acceptable practical fit.
TLI	0.918	Acceptable practical fit.
SRMR	0.047	Good absolute fit.
Scaled RMSEA	0.090	Slightly above conservative thresholds, but acceptable when interpreted with other indices and theoretical coherence.

Note. Although the scaled RMSEA was slightly above conservative cut-offs, model adequacy was evaluated using multiple criteria, including CFI, TLI, and SRMR, together with theoretical plausibility and consistency with the conceptual framework.

B. Standardized factor loadings for retained measurement-model constructs

Latent construct	Observed indicator	Standardized loading (β)
HLskill	hlmanagmnt	0.710
HLskill	hldecision	0.722
ENAB	APVENB	0.768
ENAB	InsENB	0.784
ENAB	ISENB	0.737
COMM	SDREI	0.796
COMM	GRREI	0.923
BEHAV	Practnew	1.000*

* BEHAV was specified as a single observed composite indicator in the final SEM.

C. Reliability and convergent validity of retained constructs

Construct	Cronbach's alpha	Composite reliability (CR)	AVE	Interpretation
HLskill	0.666	0.678	0.511	Acceptable for group-level analysis in an applied SEM context.
ENAB	0.793	0.807	0.585	Adequate internal consistency and convergent validity.
COMM	0.825	0.852	0.735	Strong internal consistency and convergent validity.
BEHAV	—	—	—	Single observed composite indicator; not evaluated in the same manner as multi-indicator latent constructs.

D. Discriminant validity summary

Discriminant validity was evaluated using the Fornell–Larcker criterion. Results supported acceptable separation for most retained constructs; however, the criterion was not fully met between ENAB and COMM, suggesting partial conceptual overlap in this rural context.

E. Structural path estimates in the final SEM

**Structural path**	**Standardized β**	**Unstandardized *b***	**SE**	**95% CI**	***p value***
HLskill → BEHAV	0.352	2.38	0.334	[1.722, 3.030]	<0.001
COMM → BEHAV	0.371	1.96	0.203	[1.558, 2.350]	<0.001
ENAB → COMM	0.939	1.00	0.037	[0.931, 1.080]	<0.001
ENAB → COMM → BEHAV	0.348	—	0.038	[0.273, 0.424]	<0.001

Note. Standardized β values are shown for all paths. For direct effects, unstandardized *b*, SE, and 95% CI are reported from the SEM parameter estimates. For the indirect effect, the reported CI is based on bias-corrected bootstrap resampling (1,000 iterations).

F. Additional note on interpretation

CR = composite reliability; AVE = average variance extracted. For the retained multi-indicator constructs, AVE values exceeded or were close to the conventional threshold supporting convergent validity in applied SEM. Because BEHAV was modeled as a single observed composite indicator in the final model, it is not directly comparable to the multi-indicator latent constructs in terms of CR or AVE.
